# Supplementary material for: Cognitive Behaviour Therapy for Depersonalisation Derealisation Disorder (CBT-f-DDD): Study protocol for a randomised controlled feasibility trial
Source: PLoS One. 2024 Aug 9;19(8):e0307191. doi: 10.1371/journal.pone.0307191 (PMC11315339; doi:10.1371/journal.pone.0307191)
Supplement: S1 Appendix — Spirit Figure. (DOCX) [file pone.0307191.s002.docx]

| **Data Type** | **Assessment** | | | | | | | **Assessor** | **Screening** | **T0** | **T1** | **T2** | **T3** |
| --- | --- | --- | --- | --- | --- | --- | --- | --- | --- | --- | --- | --- | --- |
| **Screening Information** | | | | |  | | |  |  |  |  |  |  |
| Contact Details | Participant Information Sheet/ Contact Information Form | | | | | | | Participant | X |  |  |  |  |
| Eligibility Screening | Screening Questionnaire (Semi-Structured Interview) | | | | | | | RA | X |  |  |  |  |
| **Personal/Clinical Data** | | | | | |  | |  |  |  |  |  |  |
| Consent | Consent Form | | | | | | | Participant/RA |  | X |  |  |  |
| Demographics | Demographic Form | | | | | | | Participant |  | X |  |  |  |
| Clinical Demographics | Demographics form | | | | | | | Participant |  | X |  |  | X |
| Intervention Allocation | Randomisation | | | | | | | Independent Statistician |  |  |  |  |  |
| **Secondary Clinical Measures** | | | | | | |  |  |  |  |  |  |  |
| Depersonalisation | CDS | | | | | | | Participant |  | X | X | X | X |
| Dissociation | DES | | | | | | | Participant |  | X | X | X | X |
| Depression | PHQ-9 | | | | | | | Participant |  | X | X | X | X |
| Anxiety | GAD-7 | | | | | | | Participant |  | X | X | X | X |
| Co-Morbidity | CIS-R | | | | | | | RA |  | X |  |  | X |
| Functioning | WSAS | | | | | | | Participant |  | X | X | X | X |
| **Intervention Training** | |  | | | | | |  |  |  |  |  |  |
| Training Course | Half-Day Workshop | | | | | | | Therapists |  | X |  |  |  |
| **Intervention Delivery** | | |  | | | | |  |  |  |  |  |  |
| Therapist Acceptability | Semi-Structured Interview | | | | | | | RA |  |  |  | X |  |
| Participant Acceptability | Semi-Structured Interview | | | | | | | RA |  |  |  |  | X |
| Supervision | CI Supervision notes | | | | | | | CI |  |  |  |  | X |
| CBT Attendance | Number of sessions attended | | | | | | | Therapist |  |  |  |  | X |
| **Intervention Fidelity** | | |  | | | | |  |  |  |  |  |  |
| Adherence | Audio recording random sample analysis of 10% of therapy sessions | | | | | | | CI |  |  |  |  | X |
|  | CTS-R | | | | | | | CI |  |  |  |  | X |
|  | CBT-f-DDD Checklist | | | | | | | CI |  |  |  |  | X |
| **Health Economics** | | | |  | | | |  |  |  |  |  |  |
| Health Service Usage | CSRI | | | | | | | RA |  | X |  | X | X |
| Quality of Life | EQ5D5L | | | | | | | Participant |  | X |  | X | X |
| **Feasibility** |  | | | | | | |  |  |  |  |  |  |
| Time of assessments | RA Timesheets | | | | | | | RA |  |  |  |  | X |
| CBT Attendance | Number of sessions attended | | | | | | | Therapist |  |  |  |  | X |
| Supervision Required | Amount and Nature from CI supervision notes | | | | | | | CI |  |  |  |  | X |
| Referrals | Number of referrals from each source | | | | | | | NHS Trusts/Unreal | X |  |  |  |  |
| Ineligible referrals | Number of ineligible referrals | | | | | | | RA | X |  |  |  |  |
|  | Reasons for ineligibility | | | | | | | RA | X |  |  |  |  |
| Reasons for not consenting | Rates and reasons for refusal to be included | | | | | | | RA |  | X |  |  |  |
| Attrition | Rates and reasons for withdrawing | | | | | | | RA | X | X | X | X | X |
