# Supplementary figures and images for: Cognitive Behaviour Therapy for Depersonalisation Derealisation Disorder (CBT-f-DDD): Study protocol for a randomised controlled feasibility trial
Source: PLoS One. 2024 Aug 9;19(8):e0307191. doi: 10.1371/journal.pone.0307191 (PMC11315339; doi:10.1371/journal.pone.0307191)

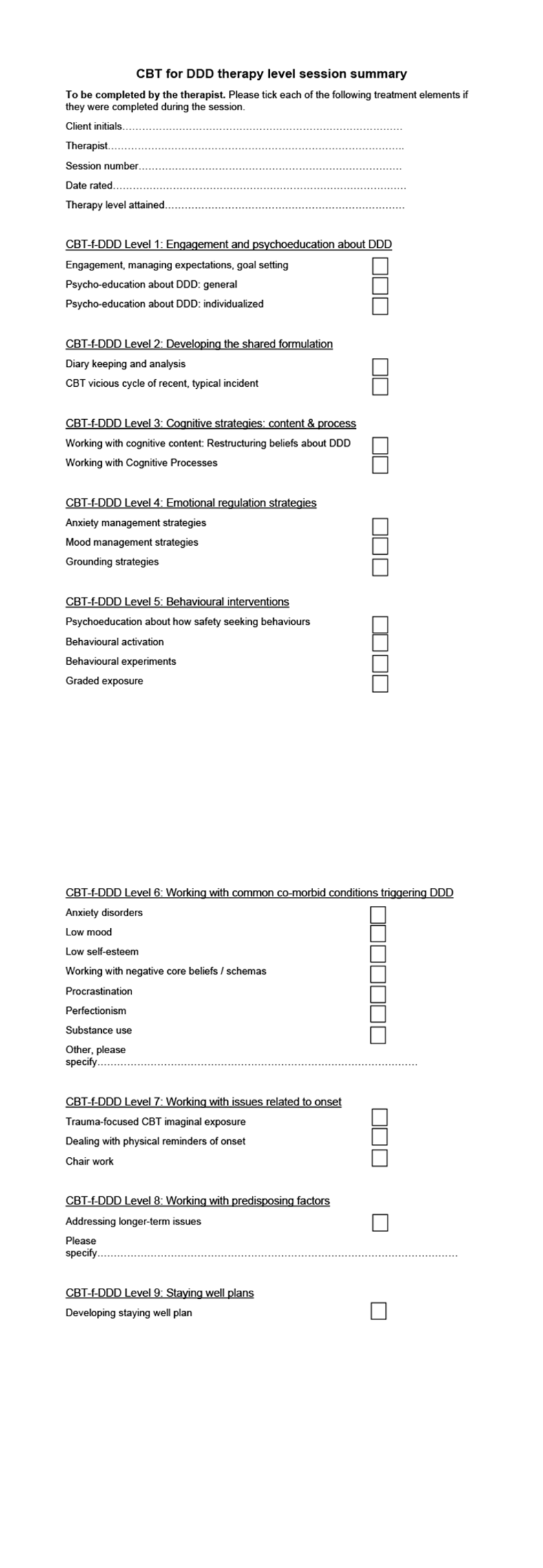

Supplement: S2 Appendix — Adapted CBT-f-DDD checklist. (TIF) [file pone.0307191.s003.tif]
