# Supplementary material for: Cognitive Behaviour Therapy for Depersonalisation Derealisation Disorder (CBT-f-DDD): Study protocol for a randomised controlled feasibility trial
Source: PLoS One. 2024 Aug 9;19(8):e0307191. doi: 10.1371/journal.pone.0307191 (PMC11315339; doi:10.1371/journal.pone.0307191)
Supplement: Attachment — Submitted filename: Reviewer Recommendations.docx [file pone.0307191.s005.docx]

"Cognitive Behaviour Therapy for Depersonalisation Derealisation Disorder (CBT-f-DDD): Study Protocol for a Randomised Controlled Feasibility Trial," it appears to be a thorough and well-structured study protocol aimed at addressing a significant gap in mental health research. The study is designed to evaluate the feasibility of a full-scale Randomised Controlled Trial (RCT) for Cognitive Behaviour Therapy tailored for Depersonalisation Derealisation Disorder (DDD), a condition that remains under-researched and under-diagnosed.

Here are are some recommendations for the editor regarding this manuscript:

Strengths:

1. Clear and Significant Rationale: The background section effectively highlights the importance of researching DDD and the potential of CBT as a treatment, establishing a strong rationale for the study.

2. Comprehensive Methodology: The study's methodology is well-designed, detailing participant recruitment, intervention procedures, data collection, and planned analyses. This thoroughness enhances the protocol's feasibility and potential impact.

3. Ethical Considerations and Transparency: The protocol includes detailed information on ethical approvals, consent processes, and data management, reflecting a commitment to ethical standards and participant safety.

4. Multidimensional Data Analysis: The plan to use both quantitative and qualitative data analysis enriches the study's approach to understanding the feasibility and acceptability of CBT-f-DDD, providing a holistic view of the intervention's potential.

\### Recommendations for Improvement:

1. Sample Size Justification: While the protocol outlines the rationale for the chosen sample size based on feasibility, further justification regarding its adequacy for preliminary efficacy analysis might strengthen the proposal. Exploring the expected effect size based on prior studies could offer additional insight into the chosen sample size.

2. Diversity and Inclusion: Given the mention of narrow population demographics in previous studies, it would be beneficial to outline specific strategies for ensuring a diverse participant pool, addressing potential barriers to participation for underrepresented groups.

3. Clarification on Control Group Treatment:*The protocol mentions that the control group will receive Treatment as Usual (TAU). Clarifying what TAU entails within the two NHS trusts and how it differs from the intervention could provide valuable context for interpreting future results.

4. Long-Term Follow-Up Plans: Although the study focuses on short-term feasibility and acceptability, discussing plans or potential for long-term follow-up could enhance understanding of the sustained impact of CBT-f-DDD, addressing whether symptom improvements are maintained over time.

5. Dissemination Strategy: Expanding on the dissemination plans to include patient and public involvement strategies might increase the study's impact. Outlining how findings will be communicated to participants, patient groups, and the wider public could foster engagement and awareness.

Conclusion:

The study protocol presents a well-considered approach to a significant gap in mental health treatment research. With minor enhancements to strengthen the rationale for sample size, ensure diversity, clarify control group treatment, consider long-term impacts, and expand dissemination plans, this protocol has the potential to significantly contribute to the field and pave the way for a larger-scale RCT.
