# Supplementary material for: Cognitive Behaviour Therapy for Depersonalisation Derealisation Disorder (CBT-f-DDD): Study protocol for a randomised controlled feasibility trial
Source: PLoS One. 2024 Aug 9;19(8):e0307191. doi: 10.1371/journal.pone.0307191 (PMC11315339; doi:10.1371/journal.pone.0307191)
Supplement: Attachment — Submitted filename: Response to reviewers.docx [file pone.0307191.s006.docx]

Dr. Muhammad Shahzad Aslam

Academic Editor

PLOS ONE

9^th^ May 2024

Dear Dr Shahzad Aslam,

**Re: Cognitive Behaviour Therapy for Depersonalisation Derealisation Disorder (CBT-f-DDD): Study Protocol for a Randomised Controlled Feasibility Trial**

Thank you for your kind email of the 25^th of^ March 2024.

We are delighted to respond to the reviewer comments raised during review and we thank you for having considered our submission. We are grateful to the reviewer for raising these interesting points which we are hopeful that we have addressed satisfactorily in this review.

We identify changes to the text we have made in the main submission here in blue for ease of reference.

We would be delighted to reconsider any further reviewer comments if necessary.

We look forward to hearing from you.

Kindest regards,

Elaine Hunter

[e.hunter@ucl.ac.uk](about:blank)

Department of Psychiatry,

University College London, UK

**Reviewer Comment**

**1. Sample Size Justification: While the protocol outlines the rationale for the chosen sample size based on feasibility, further justification regarding its adequacy for preliminary efficacy analysis might strengthen the proposal. Exploring the expected effect size based on prior studies could offer additional insight into the chosen sample size.**

**Response**

There is no formal sample size calculation, or priori power calculation. Instead, a minimum of 24 participants, 12 in each arm, has been suggested for pilot studies. This minimum recruitment strategy requires recruiting 29 eligible participants in total (encompassing a nominal 20% dropout rate). This study aims to recruit 20 participants in each arm who complete. We believe this number will give us a final sample large (and sufficiently diverse) enough to assess the practicalities of recruitment, retention, randomisation, and intervention delivery, as well as to obtain the necessary data for the statistical inferences required.

A previous naturalistic self-controlled cross-over analysis found the effect size between the treatment period and the waiting period to be 0.52 (improvement in favour of treatment). This translated into a decrease of 36 points on the CDS Scale. Using this change score, a correlation of 0.9 which was observed for intra-participant correlation, and the standard deviation of the CDS at each phase of the crossover study, we anticipated that the required sample size would be 29 participants per arm (using the ANCOVA method). Our planned recruitment of 20 per arm provides sufficient power to provide information on the magnitude, direction and precision of the treatment effect from the feasibility study to power (with sufficient precision) a subsequent definitive RCT if appropriate. (Hunter ECM, Wong CLM, Gafoor R, Lewis G, David AS. Cognitive Behaviour Therapy (CBT) for Depersonalization Derealization Disorder (DDD): a self-controlled cross-over study of waiting list vs. active treatment. Cogn Behav Ther. 2023 Nov;52(6):672-685.)

**Reviewer Comment**

2. Diversity and Inclusion: Given the mention of narrow population demographics in previous studies, it would be beneficial to outline specific strategies for ensuring a diverse participant pool, addressing potential barriers to participation for underrepresented groups.

**Response**

**Diversity and Inclusion.** Diversity and Inclusion. To increase the likelihood that the study sources a representative sample, recruitment will take place across two London mental health trusts covering six boroughs. Camden and Islington NHS Foundation Trust (C&I) is an inner London trust which had over 45,600 service users in 2020 (18). The trust covers the boroughs of Camden, Islington, and Kingston. Islington is the most densely populated local authority area in England and Wales and is among the poorest 20% of neighbourhoods in England (19). Barnet, Enfield and Haringey NHS Foundation Trust (BEH) is an outer London trust which had 146,000 service users in 2022 (20). Approximately a third of individuals across all six boroughs are residents from black, Asian or minority ethnic groups (19, 21–25). We will be recruiting from primary, secondary and tertiary care services across these Trusts.

**Reviewer Comment**

3. Clarification on Control Group Treatment: The protocol mentions that the control group will receive Treatment as Usual (TAU). Clarifying what TAU entails within the two NHS trusts and how it differs from the intervention could provide valuable context for interpreting future results.

**Response**

For some participants, the TAU will involve contact with a therapist through their mental health team or medication prescribed by their GP and/or mental health team. The TAU will vary from individual to individual but may consist of waitlist, short-term psychological therapies, or prescription of antidepressants such as SSRIs. Participants will be asked to keep a record of the type and frequency of their appointments with clinical services and treatments offered. These data will be supplemented with information provided by their key worker or through clinical note screening.

**Reviewer Comment**

4. Long-Term Follow-Up Plans: Although the study focuses on short-term feasibility and acceptability, discussing plans or potential for long-term follow-up could enhance understanding of the sustained impact of CBT-f-DDD, addressing whether symptom improvements are maintained over time.

**Response**

Unfortunately, due to the trial being funded as a feasibility study there is no scope for longer-term follow-up plans. This would be an important aspect to address if a subsequent definitive trial was funded.

**Reviewer Comment**

5. Dissemination Strategy: Expanding on the dissemination plans to include patient and public involvement strategies might increase the study's impact. Outlining how findings will be communicated to participants, patient groups, and the wider public could foster engagement and awareness.

**Response**

Dissemination of the findings will take place within Unreal, NHS partners, University College London (UCL), and the wider academic/CBT community. This will be achieved through conference presentations, and written peer-reviewed publications. Participants will have the option of requesting study results upon completion by ticking a box in the consent form. Unreal as a co-applicant on this study will assist the dissemination of materials to the lay public across various platforms. There will be a plain English summary published on the charity website and the results of the study will be included in their charity newsletter which is circulated to 1100 recipients (33). Additionally, a plain English thread will be posted on their X account to reach both wider academic audiences and clinical populations.

**Reviewer Comment**

6. Adjust citation method to adhere to journal guidelines.

**Response**

This has been adjusted to adhere to journal guidelines.

**Reviewer Comment**

7. Clarify the term "talking therapy" and specify included treatments like counselling or CBT.

**Response**

The wording has been adjusted to psychological therapies.

**Reviewer Comment**

8. Include the number of sessions in the initial CBT study on DDD.

**Response**

This was included in text:

There have been two uncontrolled studies of consecutive referrals of patients with DDD to a tertiary specialist clinic ^10,11^. In the first of these, 21 participants, most with longstanding severe symptoms which had not responded to medication or talking therapies, were treated with a median of 11 individual CBT sessions (IQR: 7 to 18). There was a statistically significant reduction in state DDD symptom severity (Effect Size = 0.37, 95%; [C.I: 0.12 to 0.53]) with 29% of participants no longer meeting diagnostic criteria at the end of therapy. Additionally, participants experienced a reduction in the severity of general dissociation (Effect Size = 0.34; [95% C.I: 0.10 to 0.51]). A recent analysis of outcomes of 36 participants from the same clinic has replicated these initial findings ^11^. A mean decrease in trait DDD symptoms (Mean = -33.67; [95% C.I: -45.14 to -22.20]) was observed after a median of 16 sessions of CBT (IQR: 10 to 16), with additional statistically significant reductions in anxiety and depression symptoms during the treatment period relative to the waitlist period.

**Reviewer Comment**

9. Expand literature review to cover various DDD treatments comprehensively.

**Response**

In systematic reviews of the existing evidence of treatment for DDD, the strongest evidence is for CBT that has been adapted for the specific condition, with little benefit demonstrated from a variety of medications and other therapy modalities. Wang et al.,’s (9) recent systematic review comprehensively covered both psychiatric and therapeutic modalities for treating DDD concluding that the evidence was poor, heterogenous and cannot support clinical treatment. The main treatment modalities discussed included medication primarily the use of lamotrigine and Selective Serotonin Reuptake Inhibitors (SSRIs) (11–13); neuromodulation of the temporo-parietal junction and ventrolateral prefrontal cortex through repetitive transcranial magnetic stimulation (14); and CBT methods as described in this study (15–17).

**Reviewer Comment**

10. Elaborate on the process of examining diagnostic criteria, including details on diagnostic interviews and personnel involved.

**Response**

The study will aim to recruit 40 participants, 20 participants in each arm. All participants will be aged between 16-75, living in London, and have symptoms of Depersonalisation and/or Derealisation currently meeting DSM-V (1) criteria for DDD. This will be assessed by the research assistant in their initial screening interview through asking the participant to the following two questions, which make up the diagnostic criteria as set out in the DSM-V (1):

“Over the last two weeks, how often have you had:

a) Experiences of unreality, detachment, or being an outside observer with respect to one's thoughts, feelings, sensations, body, or actions (e.g., perceptual alterations, distorted sense of time, unreal or absent self, emotional and/or physical numbing). [Depersonalisation]

0 =not at all; 1=several days; 2=more than half the days; 3=nearly every day”

b) Experiences of unreality or detachment with respect to surroundings (e.g., individuals or objects are experienced as unreal, dreamlike, foggy, lifeless, or visually distorted). [Derealisation]

0=not at all; 1=several days; 2=more than half the days; 3=nearly every day.”

The total score will be calculated as follows:

1. Total score = (a + b).
   a. Scores <3 = mild-moderate severity DDD.
   b. Scores >=3 = moderate-severe severity DDD.

Other DSM-V criteria will be assessed including intact reality testing, that the symptoms result in significant distress and/or functional impairment, and that the DDD symptoms cannot be better explained by another condition. Therefore, participants will be excluded if they have any of the following co-morbid diagnoses: psychosis spectrum disorder, substance dependence, intellectual disability, Post Traumatic Stress Disorder (PTSD), or cognitive impairment due to head injury or organic disorder. Comorbidity with anxiety and/or depressive disorders will not be an exclusion criterion.

**Reviewer Comment**

11. Provide more information about "treatment as usual."

**Response**

For some participants, the TAU will involve contact with a therapist through their mental health team or medication prescribed by their GP and/or mental health team. The TAU will vary from individual to individual but may consist of waitlist, short-term psychological therapies, or prescription of antidepressants such as SSRIs. Participants will be asked to keep a record of the type and frequency of their appointments with clinical services and treatments offered. These data will be supplemented with information provided by their key worker or through clinical note screening.

**Reviewer Comment**

12. Specify the qualitative method used for data analysis.

**Response**

Qualitative data from interviews with therapists and participants will be transcribed and analysed using thematic analysis within NVivo software (supervised by co-applicant Dr Morant). This will be guided by Braun & Clarke’s six stage process (42), and conducted collaboratively to include the perspectives of clinicians and those with lived experience within the research team. Interviews will be recorded using an encrypted device for the audio recording, and data will be stored securely within the NHS. Transcription of this audio recording will be done by a professional external agency which has an over-arching contract with UCL that covers all GDPR issues (as outlined in the collaboration agreement between UCL and C&I). These audio files will be destroyed at the end of the project.

**Reviewer Comment**

13. Discuss measures taken to compare in-person and online therapy methods, considering potential differences and limitations.

**Response**

The location of these sessions will depend on participant and/or therapist preference and can be conducted face-to-face or via videoconferencing. Meta-analyses comparing the efficacy of psychological therapies conducted face-to-face versus through videoconferencing has shown similar symptomatic improvement and client satisfaction scores across conditions (21,22). Additionally, a recent RCT examining online versus in-person CBT for anxiety conditions did not find a significant difference in outcome measures between treatment conditions(23). It is not expected that this study will find any differences between the two conditions but will collect the modality used for monitoring purposes.

**Reviewer Comment**

14. Explain the duration of treatment sessions.

**Response**

Those in the intervention arm will receive a minimum of 12 (maximum of 24) 1-hour-long CBT-f-DDD sessions.
