# Supplementary material for: Cognitive Behaviour Therapy for Depersonalisation Derealisation Disorder (CBT-f-DDD): Study protocol for a randomised controlled feasibility trial
Source: PLoS One. 2024 Aug 9;19(8):e0307191. doi: 10.1371/journal.pone.0307191 (PMC11315339; doi:10.1371/journal.pone.0307191)
Supplement: Attachment — Submitted filename: Response to reviewers.docx [file pone.0307191.s007.docx]

Dr. Muhammad Shahzad Aslam

Academic Editor

PLOS ONE

19^th^ June 2024

Dear Dr Shahzad Aslam,

**Re: Cognitive Behaviour Therapy for Depersonalisation Derealisation Disorder (CBT-f-DDD): Study Protocol for a Randomised Controlled Feasibility Trial**

Thank you for your kind email of the 6^th of^ June 2024.

We are delighted to respond to the reviewer comments raised during review and we thank you for having considered our submission. We are grateful to the reviewer for raising these interesting points which we are hopeful that we have addressed satisfactorily in this review.

To facilitate review of our submission, we have taken the liberty of listing the comments raised in the last review and appending the changes we have made in the text (where appropriate) of the proposed manuscript.

1. **The reviewer agrees with the author that calculating the sample size for a preliminary efficacy analysis is unnecessary because the study is exploratory. The sample size is too small to test efficacy, so statistical analysis should avoid hypothesis testing.**

We are delighted that the reviewer concurs with our decision not to conduct a power calculation for the feasibility study.

2. **The reviewer suggests removing the aim of characterizing the probability that the results were obtained by chance from the text. They explain that while a significant p-value might indicate results are unlikely due to chance, it does not accurately measure this probability as it is influenced by many factors.**

We have removed the section of the text which refers to characterising the probability that the results have been obtained by chance. The text now reads:

“To carry out preliminary inferential analyses of the efficacy of the therapy to check if a signal has been detected in the treatment arm versus the control arm (so as to inform a subsequent larger RCT if appropriate). These analyses may include estimating the magnitude and direction of the treatment effect as well as obtaining an estimate of the uncertainty in the estimate. “

3. **They recommend interpreting p-values with caution within the context of the study design and data. For a pilot study focused on testing feasibility, they advise against conducting hypothesis testing and reporting p-values.**

We will not report p values but instead characterize the uncertainty in the estimate of the treatment effect by declaring the 95% confidence interval and standard error of the mean.

We have identified changes to the text we have made in blue for ease of reference.

We would be delighted to reconsider any further reviewer comments if necessary.

We look forward to hearing from you.

Kindest regards,

Elaine Hunter

[e.hunter@ucl.ac.uk](about:blank)

Department of Psychiatry,

University College London, UK
